# Digital Livestock Technologies as boundary objects: Investigating impacts on farm management and animal welfare

**DOI:** 10.1017/awf.2023.16

**Published:** 2023-02-17

**Authors:** Juliette Schillings, Richard Bennett, Françoise Wemelsfelder, David C Rose

**Affiliations:** 1 School of Agriculture, Policy and Development, University of Reading, Reading, UK; 2 Animal and Veterinary Sciences, Scotland’s Rural College, Edinburgh, UK; 3 School of Water, Energy, and the Environment, Cranfield University, Cranfield, UK

**Keywords:** animal welfare, boundary objects, Digital Livestock Technologies, learning, positive animal welfare, precision livestock farming

## Abstract

Digital Livestock Technologies (DLTs) can assist farmer decision-making and promise benefits to animal health and welfare. However, the extent to which they can help improve animal welfare is unclear. This study explores how DLTs may impact farm management and animal welfare by promoting learning, using the concept of boundary objects. Boundary objects may be interpreted differently by different social worlds but are robust enough to share a common identity across them. They facilitate communication around a common issue, allowing stakeholders to collaborate and co-learn. The type of learning generated may impact management and welfare differently. For example, it may help improve existing strategies (single-loop learning), or initiate reflection on how these strategies were framed initially (double-loop learning). This study focuses on two case studies, during which two DLTs were developed and tested on farms. In-depth, semi-structured interviews were conducted with stakeholders involved in the case studies (n = 31), and the results of a separate survey were used to complement our findings. Findings support the important potential of DLTs to help enhance animal welfare, although the impacts vary between technologies. In both case studies, DLTs facilitated discussions between stakeholders, and whilst both promoted improved management strategies, one also promoted deeper reflection on the importance of animal emotional well-being and on providing opportunities for positive animal welfare. If DLTs are to make significant improvements to animal welfare, greater priority should be given to DLTs that promote a greater understanding of the dimensions of animal welfare and a reframing of values and beliefs with respect to the importance of animals’ well-being.

## Introduction

The development of smart technologies is viewed as a key response to the increased concerns around sustainability (Walter *et al*. 2017). In the context of population growth and rising demands for livestock products, farm animal welfare is gaining attention (European Commission 2016). However, ensuring good animal welfare, improved productivity, and minimal impacts on the environment of livestock production systems is ever more difficult as a decrease in the number of farmers makes attending to the needs of an increasing number of animals more challenging (Eurostat 2020). It is for these reasons that Digital Livestock Technologies (DLTs), such as Precision Livestock Farming (PLF) technologies, have gained particular interest, as they allow improved monitoring of animals. By tracking changes in animal behaviour or physical parameters, DLTs can help detect health and welfare compromises at early stages, thus facilitating farmers’ work and giving them better control over livestock management (Berckmans 2014; Kling-Eveillard *et al*. 2020). There are different forms of DLTs, including wearable sensors (e.g. collars, leg- or ear-tags), digital cameras or microphones, that can detect, for example, heat or lameness in dairy cattle, respiratory health in pigs, or environmental parameters in poultry farms. These technologies present many benefits; from minimising risks of diseases or injuries to reducing costs and improving animal productivity and health and welfare in a variety of production systems (Schillings *et al*. 2021).

Although the benefits of DLTs in relation to animal welfare are often promoted, the extent to which DLTs can help improve animal welfare is still unclear. As Dawkins (2021) suggests, this is likely to depend on how animal welfare is defined, how much it will be considered in technology developments, and whether DLTs will be able to deliver on their promises. Animal welfare is a complex notion that can be understood differently by different people. Such divergence of perception can have a range of implications, as reaching a consensus when defining ethical ways of keeping animals becomes challenging, and initiatives to improve animal welfare may fail (Fraser 2008; Dawkins 2021). Whilst, historically, reducing harm and negative experiences have often been the focus of animal welfare science, the importance of positive animal welfare, which emphasises the capacity for animals to experience positive affective states and to live good lives, is increasingly highlighted (Boissy *et al*. 2007; Mellor & Beausoleil 2015; Lawrence *et al*. 2019).

Determining how DLTs may impact farm animal welfare should thus not only be based on their capacity to better detect health and welfare compromises. It should also focus on whether tools can foster learning and a shared understanding of the notion of animal welfare, particularly on the importance of promoting positive animal welfare. We suggest that this capacity is likely to depend on the ability of DLTs to act as boundary objects. Boundary objects are defined as “*objects that are plastic enough to be adaptable across multiple viewpoints, yet maintain continuity of identity*” (Star 1989; p 38). They can be ‘material’ objects or theories and concepts which, while sharing common definitions and goals, may be interpreted or used differently by different actors (Star 2010). In their study, Jakku and Thorburn (2010) conceptualise Decision Support Systems (which include DLTs) as boundary objects through which different actors can collaborate and co-learn during their development. By opening discussions and collaborating, stakeholders can increase their understanding of a specific issue, even when holding diverse views about it.

Whilst the concept of boundary objects has not, to the authors’ knowledge, been applied in the context of farm animal welfare, studies have investigated the potential of boundary objects to facilitate discussions, knowledge-sharing and impacts on management practices in the context of sustainable farming (Morris *et al*. 2020; Zinngrebe *et al*. 2020; Hochman *et al*. 2021). Morris *et al*. (2020), for example, found that using boundary objects (a simulation tool and board game) fostered learning and changes in livestock management practices by supporting perspective sharing among stakeholders on strategies to transform livestock production. Similarly, a study focusing on the participatory development of a Decision Support System to improve nitrogen fertiliser use in sugarcane production also found that, by acting as boundary objects, the system allowed stakeholders to explore management strategies and co-learn, resulting in changed perceptions of local sugarcane production systems and their management (Thorburn *et al*. 2011).

By acting as boundary objects, the use of DLTs could thus facilitate discussions between farmers and other stakeholders (e.g. advisors, technology developers, consumers, or retailers), and foster learning around the notion of animal welfare. This could, in turn, lead to changes to management practices with varying impacts on animal welfare. Indeed, learning plays a key role in decision-making and changed farm management practices (Kilpatrick & Johns 2003; Leeuwis 2004). The main sources of learning for farmers were found to be mostly unstructured and informal, such as through observation and experience, and through social and business networks and interactions with peers or advisers (Kilpatrick & Johns 2003). As Kilpatrick and Johns (2003; p 154) note: “*[i]nteraction allows farmers to compare views on how information could be applied to their own situations and to test each other’s values and attitudes toward making changes as a result of the information.*”

Different types of learning can, however, lead to different outcomes. Leeuwis (2004) distinguishes between regular and architectural innovations, the former involving learning about how to make improvements within the boundaries of basic cognitive assumptions and principles (such as norms, goals and values). Such learning can be referred to as ‘single-loop learning’ (Argyris & Schön 1978): the process of modifying and improving strategies as a result of detecting errors. In contrast, architectural innovations involve a questioning of these assumptions and principles and a shift in how strategies are framed (double-loop learning). In other words, single-loop learning relates to the ‘know-what’ and ‘know-how’ dimensions of knowledge, whereas double-loop learning involves a ‘know-why’ dimension (Reed *et al*. 2016a). The impacts of DLTs on animal welfare may thus be greater if they can foster a deeper reflection on farmers’ underlying values and norms (double-loop learning), since these influence farmers’ motivations to seek more knowledge about animal welfare or to engage in management practices that can support its improvement (te Velde *et al*. 2002; Vigors & Lawrence 2019).

Little is currently known about the potential of DLTs to act as boundary objects and the extent to which they can foster learning around the notion of animal welfare, and what the consequences would be for farm management practices. The aim of this study was thus to explore these topics using the concepts of boundary objects and single- and double-loop learning, and to discuss the potential impacts on farm management practices and their significance to the enhancement of farm animal welfare. The study focuses on two case studies during which two different DLTs were tested. The results of a survey were used to gain further knowledge on the impacts of DLTs on farm management practices and animal welfare.

## Materials and methods

In this study, qualitative and quantitative approaches were combined to obtain a wider and more in-depth understanding of the possible impacts of DLTs on farm management and animal welfare. The use of case studies allows one to obtain an in-depth understanding of a number of cases in their real-world context, with the hope that the result sees learning about real-world behaviour and its meaning (Yin 2011). Combining case studies with a survey can help procure a more complete picture of the answers to our research questions.

### Ethical considerations

Since the study involved interviews with human subjects and the dissemination of a survey, the research project has been reviewed according to the procedures specified by the University of Reading Research Ethics Committee which granted ethical clearance for this project. Participation in this research was completely voluntary. The research did not require the collection of information that may have been considered sensitive in terms of confidentiality, or that may have caused personal upset. It did not involve elements of deception, and participants were offered a guarantee of anonymity and secured data storage, and the possibility to withdraw from the study at a date specified in information sheets (provided to interview participants and at the start of the survey).

### Case study descriptions

In-depth, semi-structured interviews were conducted by JS with stakeholders from both case studies (n = 31), using topic guides (for an example, see Appendix 1). This method allows generation of large amounts of detail about participants’ experiences, whilst allowing the discussion to be guided to address our research questions.

#### Case study A: Cattle mobility and body condition scoring

A camera system to monitor Body Condition Scores (BCS) and mobility in dairy cattle was developed and tested on eleven pilot farms in the UK, nine of them trialling the technology in the context of a farm assurance programme. Body condition and mobility are important factors that can influence animal health and welfare, as well as productivity (Whay & Shearer 2017). These measures are usually undertaken by humans which poses the risk of introducing biases and errors. Automating these measures was thus seen to reduce these risks while allowing farmers to spot any changes in conditions early (Silva *et al*. 2021). The system scores cows each time they pass beneath the camera, which is placed at the exit of the milking parlour.

Two rounds of interviews were conducted. The first involved all eleven farmers before they were able to use the technology. Interviews were held between August 2020 and May 2021 for 46 min duration on average, using the phone or video conference software (e.g. Microsoft Teams®) due to COVID-19 pandemic restrictions. The discussions involved health and welfare management, general cattle welfare and farmers’ use of DLTs. The second round of interviews was conducted with nine of the eleven farmers, as one farmer had sold their cows during the project, whilst another was not able to install the technology. Two technology developers and a stakeholder involved in the quality assurance programme were also interviewed. These were held for 53 min duration on average, using the same platforms, between March 2022 and April 2022. The gap between the first and second rounds of interviews is explained by technical issues in addition to external challenges such as the COVID-19 pandemic, which delayed the project by several months. It also meant that the system was fully operational for only two participating farmers at the time of the interviews. The results and discussions are thus based primarily on farmers’ expectations and perspectives; except where specified for those farmers having used the technology. Themes addressed during the second round included changes to management practices and welfare, learning and impacts on attitudes towards animal welfare.

#### Case study B: Smartphone application

A smartphone application was developed by a UK research institute, and licensed and trialled by a UK retailer, to allow farm assessors to assess animals’ emotional well-being by scoring their expressive demeanour. The application can be used in different livestock farming systems, including cattle, poultry, pigs, sheep, goats, and salmon. For each species, a list of 15–20 descriptive terms balanced for positive and negative expressivity (e.g. relaxed, joyful, tense, anxious) was developed participatively by the retailer’s supply chain stakeholders (e.g. farmers, farm assessors, veterinarians), based on participants’ experience and on a discussion of videos showing animals in a variety of environments. These terminologies were inserted into the application. When visiting a farm, after observing the expressive demeanour of animals on that farm, farm assessors would score each descriptor on sliding scales. The application then integrates these scores through multivariate statistical analyses and produces a graph locating visited farms in overall patterns of emotional well-being. This graph can be used by assessors to make comparisons between farms and to discuss with individual farmers how emotional well-being on their farms may be managed or improved.

The same topics as in case study A were discussed with participants during a single round of interviews held in May 2022, using the same platforms. These lasted 50 min on average. The lead researcher, who developed the method on which the application was based, provided contact email addresses for 21 stakeholders of the retailer’s supply chains who were involved in trialling the application and who were willing to be contacted by JS. From the 21 people contacted, 16 stakeholders covering different species subsequently agreed to be interviewed, including farmers, farm assessors, supply chain directors and others involved in the project (e.g. co-ordinators, managers). An interview was also conducted with the lead researcher.

Quotes from case study participants were used to support statements in the *Results* and *Discussion.* For case study A, we identified farmers as ‘farmer 1’ to ‘farmer 11’, and developers as ‘developer 1’ and ‘developer 2.’ For case study B, stakeholders were identified as ‘participant 1’ to ‘participant 16.’

### Qualitative data analysis

Interviews were recorded using a smartphone application or software recording options (e.g. Microsoft Teams®). The interviews were transcribed *verbatim* by the first author, allowing better familiarisation with the data (Braun & Clarke 2006). The data were then analysed thematically using a qualitative data analysis software (NVivo 12) for coding. Analysis was guided by methods from Braun and Clarke (2006) and Ritchie *et al*. (2014). First, an initial thematic framework was produced, using a series of themes and sub-themes which covered the aims of the study. The data were then coded into these themes, with new ones emerging throughout the coding process. The data were then sorted, and each theme was reviewed, sometimes resulting in the deletion, or merging of themes. Finally, data summaries were produced for each theme, helping to uncover key elements and underlying dimensions that guided data interpretation.

### Farmer survey

To gain further insights into how DLTs could change management practices independently from the case studies described above, we conducted a farmer survey focused on the dairy sector and Precision Livestock Farming technologies (the latter of which are DLTs that can monitor parameters in real-time, automatically and continuously). We focused on this area because it gave us access to a wide range of commercially available technologies (extending the survey to all farmed species and types of technologies would have made analysing the data less manageable). The survey was created using Qualtrics (Qualtrics XM Software®, Provo, UT, USA). The survey was distributed online using the authors’ networks and relevant organisations and institutions, as well as on social media and farming forums. To increase the sample size, the survey was also sent by post to 250 dairy farmers in the UK. Postal addresses were found using the UK Government list of Registered Dairy Establishments (as at 1 August 2021 to 1 November 2021). As the list only provided partial addresses, full addresses were obtained using the search engine Google. Addresses from the list were selected randomly. The survey was piloted online with five dairy farmers prior to distribution, and changes were made based on farmers’ comments. Survey responses were anonymous, and an incentive of £1 was donated to the Royal Association of British Dairy Farmers (RABDF) per completed response.

A total of 33 questions could be answered by respondents, although this number varied depending on respondents’ choices (for the questionnaire, see Appendix 2). The survey was developed as part of a wider study, therefore only results relevant to this study will be discussed. Eighty-six online and 59 postal surveys were completed, leading to a total of 145 responses. Sixteen respondents who indicated using DTLs submitted partial responses which were taken into account in our results. Some respondents left certain questions unanswered. Thus, N is used throughout to describe the total number of respondents, and n the number of respondents to a specific question. Descriptive statistics were performed on the survey data using the Qualtrics® platform and Microsoft Excel®.

## Results

The interviews conducted for both case studies indicated that the DLTs used had the potential to act as boundary objects. In this section, we explain how DLTs facilitated connections between stakeholders (e.g. advisors, consumers, and producers) and which type of learning they generated, using the concepts of single- and double-loop learning. Whilst both DLTs could facilitate connections, they differed in the type of learning they fostered.

### Facilitating connections

In both case studies, it was reported that technologies could indeed facilitate discussions between stakeholders such as farmers and farm advisors (e.g. vets or nutritionists), as well as help bridge the gap between producers and consumers. Participants from case study A mentioned that using the camera, which enables constant and automatic monitoring of BCS and mobility, could help advisors tailor their advice based on the data generated; helping farmers in decision-making to boost productivity and improve animal welfare. As one developer said:

*From those two metrics, vets and farmers can derive a lot of information and understand what actions they should take to ensure productivity doesn’t decrease and ensure the welfare of the animal doesn’t get worse* [Developer 1].

Similarly, participants from case study B believed that the use of the application had the potential to connect stakeholders such as farmers, farm assessors or vets, helping to provide better insight into what happens on farms. As one participant said:

*We very much want the farmers to be engaging with it and to be able to get a better insight into what’s going on, on their farms. And again, by having the vets do it; to engender those discussions between the vet and the farmer and in turn those discussions between the independent assessor and the vet and the farmer* [Participant 1].

The application was also generally seen to open discussions between farmers on their management practices, encouraging them to discuss with other farmers what they have been doing and to identify possible improvements based on their scores. As one participant said:

*It almost has that level of friendly competition […] if perhaps you’re not in the top end […] then you want to be asking your colleagues ‘What are they doing? How can you improve that?’* [Participant 7].

The application was also considered a useful conversation tool by participants, as it also enabled actors to articulate what they were already thinking internally. One participant, for example, mentioned how the application could help put “*words on feelings and thoughts.*” As another participant said:

*The proof of the pudding is that you talk to vets and other experts that have used this tool and it very much reflects what they see independently […] They would go on-farm and […] have that feeling internally without being able to express or articulate it. Actually, the application almost always will merely reflect precisely what you’ve been thinking* [Participant 1].

Another benefit of both technologies was their ability to bridge the gap between consumers and producers. In case study A, participants mentioned that by being able to provide evidence on claims about animal welfare, consumer trust in the farming system could be improved. As the stakeholder working for the farm assurance scheme organisation said:

*We need to have that detail if we are ever challenged on the claims we’re making, we want to be transparent and truthful in everything we do, so these technologies help us to have that integrity.*

Bridging this gap was also considered a benefit of application (B). As a participant said:

*Often, there’s quite a disconnect in this country […] The farmer has very little understanding of what’s important to the shopper, and the shopper, very little understanding of where food is being produced. So, they have a job to try and bridge that gap too* [Participant 15].

On the biggest value of this application, the same participant added:

*I think, getting the message across to consumers […] to say we care about the animals in our farming systems […], that it is not just intensive, faceless, big business: it’s about real people and feelings and emotions.*

Most participants from case study B also highlighted the ability of consumers to relate to the descriptive terminologies used to generate scores in the application. They saw this as a non-specialist language that consumers can easily visualise and understand, eg terms like ‘content’ and ‘distressed’ as opposed to health metrics such as mastitis in dairy cows. It was also considered a way to demonstrate to consumers that the emotional aspects of animal welfare, in addition to minimising negative experiences and promoting good health, were taken seriously. In the same way, conversations about positive animal welfare could also be facilitated with farmers through the application. Reporting on a participant’s experience, the lead researcher said:

*One person said […] when they come on a farm visit and have quite a few assessments to make […] and they show* [the application] *to the farmer; it just opens up a conversation about something that the farmers aren’t necessarily used to talking about.*

Both technologies thus have the potential to facilitate connections between stakeholders, helping farmers in decision-making and helping consumers make better-informed choices. In case study B, the application can also open discussions on other dimensions of animal welfare (e.g. positive animal welfare).

### Learning outcomes

By connecting different stakeholders, DLTs have the potential to better inform management and foster learning and increased knowledge around animal welfare. This could have important welfare implications, although the extent of these impacts is likely to depend on the type of learning generated.

#### Single-loop learning

In case study A, the technology is designed to allow farmers to detect changes in lameness and body condition more precisely and more frequently than the human eye can, especially where large numbers of animals are involved. This allows farmers to act at an early stage, preventing, for example, more severe cases of lameness, which can develop if slightly or moderately lame cows are not detected. Using constant, unobtrusive monitoring and spotting subtle changes, farmers can minimise potential treatment costs and lower milk output as well as labour (e.g. by spending less time monitoring animals by eye). One farmer having used the camera mentioned how they learned to make better use of their time. They said:

*What it has probably done is made me have more use of my time, you know, it’s pinpointing things down and getting them fixed* [Farmer 8].

They also noted the benefit of not having to move cows to score them, which enhances productivity and welfare:

*The less you can move the cows you’ll find the better yield is because […] the less you’re moving them, the less you’re upsetting their natural behaviour and therefore it’s probably helping drive milk yield* [Farmer 8].

Others mentioned how the system could help farmers “*do a better job*” by being more proactive and better organised. One of them said:

*The camera has great benefits because it’s picking up differences over a certain period of time […] so we’ll be able to see any changes before the human eye can; we’ll be picking things up more proactively* [Farmer 3].

In turn, this could help improve performance through better animal health and welfare. As one farmer said:

*The technology can tell us that she’s going lame before you can visibly see it […] in order for us to treat the animal promptly and hopefully prevent a problem from going considerably worse* [Farmer 1].

A farmer that was able to use the camera also noted the advantage of being less able to ignore cows that were slightly lame, which they would sometimes do in the past:

*I think the biggest change using the camera is […] pulling out those cows that aren’t lame but need a trim. Sometimes it was easy to put off like, you might notice she’s got long feet but she’s not too bad, she’ll do. But I think the fact that it’s actually physically on our computer […] you probably think, alright, it’s on paper […] I really ought to be getting that done* [Farmer 8].

Similarly, another farmer using the camera mentioned looking at slightly lame cows more attentively:

*You do look harder at them. If it’s flashing up as a problem or as slightly lame, then you concentrate harder on that one* [Farmer 2].

In case study B, the use of the application was also seen as a possible way to improve performance on farms by being able to link data on emotional expressivity to other welfare metrics, facilitating a better understanding of where improvements might be needed or possible. For example, links might be made with animal performance, encouraging farmers to adapt management of their farm (e.g. by improving the animals’ environments). A participant involved in the pig supply chain said:

*We could see patterns starting to build and if we could then marry that up with a reason […] you might then be able to […] work out if there’s an issue […] Then you could start making some changes to increase the welfare and the benefit to those pigs* [Participant 3].

The application was also seen as a way to gain knowledge about the importance of practices that have the potential to enhance animal welfare, such as grazing or the use of enrichment. As a participant said:

*If you come from a farming perspective: actually, I want my cows to be in the best place emotionally as possible, and I have got that evidence from down the road that those cows are in a really good place and they’ve got the following enrichment opportunities, then perhaps I can adopt those. So, it’s an opportunity for education for farmers as well as everybody else* [Participant 1].

Being able to compare between farms was another mentioned benefit of the application, as it allowed an understanding of where improvements are possible based on evidence. The application was indeed also considered a useful tool for benchmarking. As a participant said:

*Farmers consistently scoring lower than other farms, it might be kind of understanding why that is and what could change; what other farms are doing that’s giving them greater scores and improving those systems* [Participant 2].

#### Double-loop learning

The possibility to improve monitoring and livestock management strategies was observed in both case studies. They differed, however, in the extent to which participants had learned to question those strategies. A farmer from case study A mentioned how the use of the camera system led them to question the level of welfare on their farm in relation to lameness prevalence, which is an issue we mentioned earlier. On this, they said:

*The couple of times that I’ve been onto the website […] and thinking, oh, maybe that’s more than I expected […] It has made me think about the welfare of the cows. Is it as good as I think it is? […] Unfortunately, as farmers, you can get a bit blinkered to your own farm* [Farmer 9].

However, double-loop learning outcomes in case study A were somewhat limited. When asked about whether the use of the camera had influenced their understanding of animal welfare or their approach to the concept, one user clearly stated not having learned much:

*To be fair, no. Lameness is lameness. Everyone is aware of what it is and the problems it has. So no, it doesn’t change that way* [Farmer 2].

Another farmer said their perception of welfare did not change following the use of the system, particularly as they were already conscious of it. They said:

*I don’t know if it changed my perception around animal welfare because I think it’s always been fairly high up on the list […] especially with lame cows, because public perception-wise, that’s the easiest thing to pick on* [Farmer 8].

In contrast, case study B participants mentioned how the use of the application made them think more actively about animal welfare, particularly in relation to animal emotions. Indeed, the method encouraged users to take the time to observe animals and take a close look at how they expressed themselves, both in relation to other animals and the environment which stimulated users to ask ‘*why*’ animals were behaving a certain way. As one of them said:

*We’re certainly far, far more dialled into watching a behaviour as an expression and not just its natural behaviour or its aggressive behaviour or whatever else. Actually, no, why are they doing that? What are they feeling in order to be doing that behaviour? […] What is it that’s behind that? So that’s the big step change really* [Participant 15].

On this, another participant added:

*It might give them time to think about ‘why are they?, ‘what’s going on here?’ That’s why I think the app’s got a useful position and a useful time to play in the farmers’ day* [Participant 3].

On taking time to observe animals, a participant also said:

*I think we can almost ignore what the actual outcome is, it’s more a means of encouraging the stockman to have a look at his flock* [Participant 12].

The application also made users think about the animals’ perspectives and look at them in different ways. A shift in attitudes and approaches to welfare was observed, with a stronger focus on animals’ emotional states, as opposed to a sole focus on physical parameters. One participant explained that they had not considered positive welfare in the past, but that the use of the application led to a change in perspective:

*I didn’t necessarily really look at the cows and think how happy they were in their environment and how comfortable they seem […] but yeah, it’s definitely got me looking at them in a different way* [Participant 13].

The application had the potential to drive change and encourage improvements to work towards positive animal welfare. A participant explained how they experimented with different lighting conditions, positions of enrichment bales and perching in poultry farming, and based their judgement on observing changes in the animals’ emotional expressivity. They were then able to advise farmers they were working with to adapt management accordingly. On lighting conditions, they said:

*When I was doing the assessment, I noticed that the behaviour of the birds was affected by light intensity […] The behaviour of the birds* [at the minimum legal requirement of lighting intensity – 20 lux] *was very different to birds at 30 lux light intensity. So, there is a couple of farms […] I got them to upgrade their lighting system, and I can see a positive change in the birds’ behaviour already* [Participant 14].

Through developing the application and discussing with other stakeholders, participants also reported getting a wider knowledge and understanding of animal expressivity and differences between species. Making use of a wide range of terms to explain subtle differences, the application was seen as a tool to train farm staff to look at animal emotions. As a pig farmer said:

*I think it would be quite a good training way, engaging people to actually look at the pigs as animals […] Unfortunately, there are farms where they don’t have that level of empathy […], so I think from a training point of view, that would be good* [Participant 6].

The potential of the application to make users think differently and promote reflection was further emphasised by other participants. One of them, for example, mentioned how the application was about changing producers’ mentality. They said:

*The biggest benefit that I saw from day one is not about the detail and the data, it’s about changing the mentality of the producer to think in terms of the feelings of that animal* [Participant 15].

Others mentioned how using the application was ‘*thought provoking*’, helping focus the mind, promoting the subconscious and making them think outside the box. One of them mentioned how using the method became part of their routine; constantly monitoring animals in their heads whilst doing routine jobs. They said:

*It doesn’t matter what job you’re doing in life; you always need to challenge your thoughts on what you’re doing, and there are all those things you can do better. And I think using the app, it challenges your thoughts* [Participant 14].

The use of DLTs thus had important learning outcomes and promoted changed management practices in both case studies. This ranged from improved strategies to reduce lameness with more efficient monitoring and early treatment in case study A, to changing attitudes to observing animal expressivity in case study B, leading farmers to pause and reflect on ‘why’ animals were behaving in certain ways, and then finding ways to adapt management in order to encourage positive expressivity.

### Possible challenges to welfare improvements

Some possible barriers to welfare improvements were identified through the case studies. An important challenge was that of changing farmers’ mentality. In case study A, some farmers mentioned not wanting to have a look at the data, as it was telling them something negative. As a farmer said:

*You know, as a farmer, if it tells you something that makes you feel a bit depressed, i.e. you’ve got really lame cows. Then you’re just a bit like, oh, I don’t know if I want to look at it* [Farmer 9].

Perception of lameness levels is a commonly identified issue in dairy farming, and this was something that one of the system’s developers learned during this experience. They said:

*I think it’s highlighted that farmers are not always very good at perceiving the level of lameness or body condition […] I think a lot of farmers are perhaps more optimistic of their scores than what’s actually going on* [Developer 2].

Some farmers from case study A also believed the system would add more work, especially where they already had their routines, or when they considered that lameness was not an issue on their farm.

Farmer mentality was seen as a challenge by some participants from case study B, who mentioned that farmers could be sceptical about the application. As one of them said:

*Seeing somebody coming in and putting some sliders on a mobile phone and then coming up with an assessment […] they’d just look at it, thinking, well, I could’ve told you that, I know what these birds are like* [Participant 12].

On this, another participant said:

*In a way, I have to go into their farm to ask whether their cattle are happy or not. It’s almost a little insulting. I would be insulted if somebody came to my house and said, right, I’m gonna get this application on my phone and I’m gonna determine whether your dogs are happy or not* [Participant 10].

Other participants also struggled with the qualitative nature of the method, particularly in the context of animal welfare assessments, which often rely mostly on quantitative criteria. Some participants found it challenging to use terminologies that included terms such as ‘happy’ or ‘depressed’ to describe animals and wondered whether this could be interpreted as being anthropomorphic. The lead researcher recognised that making anthropomorphic mistakes is a risk but emphasised that the use of qualitative descriptors is not by definition anthropomorphic. They said that the value of the method relies on observations made by skilled, experienced assessors, and that rather than imposing mechanistic criteria, the answer to the risks of anthropomorphism is more training. As they said:

*There’s no point trying to make* [the method] *more credible by trying to objectify and mechanise and instrumentalise it, which is what so many scientists think is required to make it objective. But you kill it off if you do that.*

### Survey results on management practices

**Figure 1.**
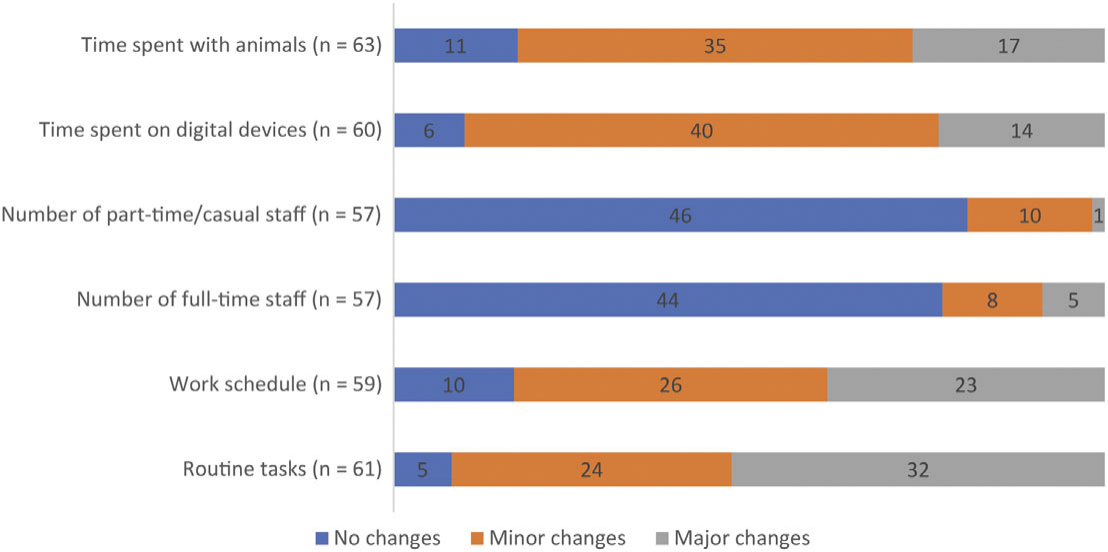
Distribution of management changes observed after using PLF technologies.

Whilst results from the survey do not allow inferences to be made on the extent to which PLF technologies may act as boundary objects, they allow exploration of the extent to which they may impact farm management and animal health and welfare. This partially helps us to consider what type of learning may have resulted from using the technology. When asked about changes to management routines due to using PLF technologies, 92% of survey respondents indicated making changes to routine tasks, with 52% observing major changes. Eighty-three percent also observed changes to their work schedule, with a majority observing minor changes (44%) (Figure 1). Most respondents did not experience changes in terms of numbers of full- and part-time staff (77 and 81%, respectively). Most, however, experienced changes in the time they spent on digital devices (90%) and with animals (n = 63; 82%), with 23 and 27% observing major changes, respectively.

Rating on a Likert scale from 1 (substantially decreased) to 5 (significantly increased), most respondents indicated that the time spent visually or manually assessing animal health and welfare had somewhat decreased (n = 67, Mdn = 2, IQR = 1). However, most chose not to rely on the data completely; as they manually or visually verified the data collected most of the time (n = 66, Mdn = 4, IQR = 2; on a Likert scale from 1 [never] to 5 [always]).

This decrease did not seem to impact the human-animal relationship (HAR) as, rating on a Likert scale from 1 (substantially decreased) to 5 (significantly increased), most indicated that human contact with cows remained about the same (n = 67, Mdn = 3, IQR = 0), and that the relationship between stockpeople and the herd did not change since using technologies (n = 67, Mdn = 3, IQR = 1). However, 39% of participants did report an improved relationship (6% indicated a ‘much better’ relationship and 33% a ‘somewhat better’ relationship).

In terms of impacts on animal welfare, most respondents indicated that the use of PLF technologies had helped make the parameters they were designed to monitor ‘somewhat better’, eg improved heat or lameness detection (n = 59, Mdn = 4, IQR = 1), when rating on a Likert scale ranging from ‘much worse’ (1) to ‘much better’ (5). The same was observed when asked about respondents’ perceptions of overall animal welfare levels since implementing PLF, as most indicated that it was somewhat better (n = 61, Mdn = 4, IQR = 1), with 20% of respondents perceiving ‘much better’ welfare.

**Figure 2.**
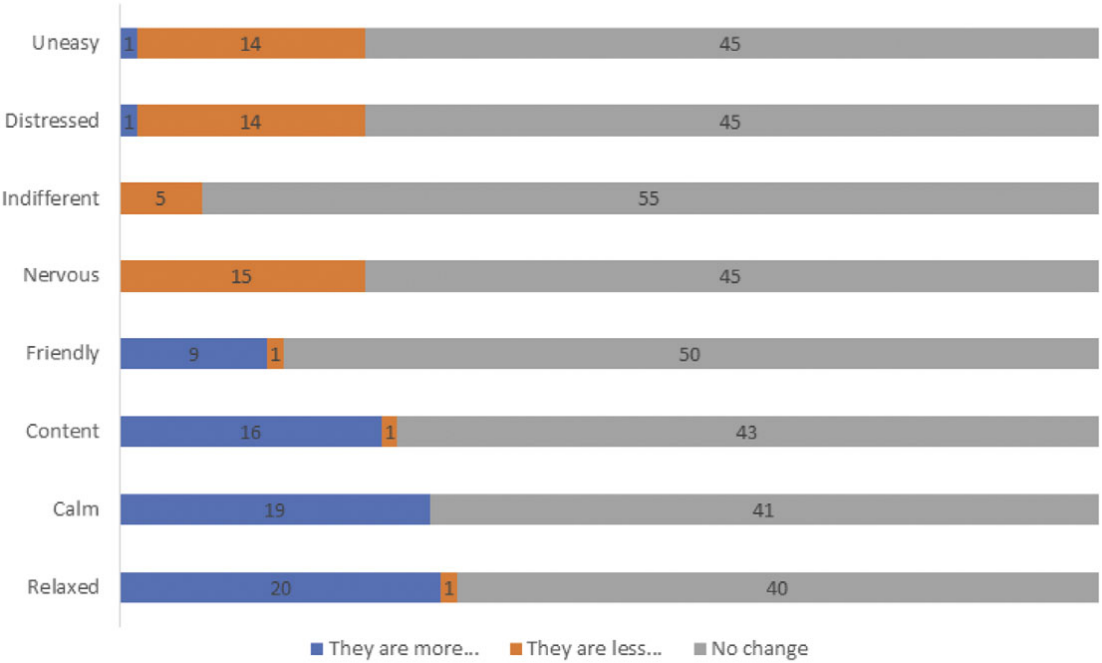
Changes observed in animal behaviour inspired by a fixed list of descriptors used in the Welfare Quality® protocol for dairy cattle (2009).

Finally, respondents were asked to indicate whether they observed changes in their livestock following the use of PLF based on a list of eight descriptors, using the options ‘they are more…’, ‘they are less…’ or ‘no change’, associated with the following descriptors: ‘relaxed’, ‘calm’, ‘content’, ‘friendly’, ‘nervous’, ‘indifferent’, ‘distressed’ and ‘uneasy’ (Figure 2). The descriptors used in the survey were independent from those used in case study B described above and were inspired by a fixed list of qualitative descriptors that can be found in the Welfare Quality® protocol for dairy cattle (Welfare Quality® 2009). Whilst most respondents indicated no change, some indicated that they believed their cows were more relaxed (33%), calm (32%), content (27%), friendly (15%) and that they were less nervous (25%), distressed (23%) or uneasy (23%).

## Discussion

The welfare of farmed animals is highly dependent on human decisions (Boivin *et al*. 2003), thus the extent to which DLTs may help improve welfare will depend on the way they affect management practices, and whether important aspects of animal welfare (e.g. the HAR and animals’ ability to live positive experiences) are considered and improved. Findings from the present case studies indicate that, by acting as boundary objects, DLTs can help inform management by facilitating connections between different actors and fostering learning to improve productivity and better respond to citizens’ concerns around sustainability and animal welfare issues. Indeed, the tools could help improve communication and transparency relating to animal health and welfare, or to spot animals whose welfare may be compromised more efficiently.

The technology from case study A, for example, could help farmers be more organised and proactive in relation to lameness, which is an important welfare issue (Whay & Shearer 2017). In a study conducted on European organic dairy herds, it was found that overall lameness prevalence reached 18%, with some farms reaching 79% (Sjöström *et al*. 2018). This can be linked to the fact that farmers often underestimate lameness prevalence and the implications for productivity, making it a barrier to reducing lameness prevalence (Leach *et al*. 2010). Farmers often delay the treatment of less severely lame cows due to limited staff resources, having to balance with other farm priorities, or not understanding the value of prompt, early treatment (Horseman *et al*. 2014). Thus, the use of a camera could have important welfare implications if it results in the early treatment of lame cows, and a stronger focus on less severe cases as a method of prevention.

Both technologies also had the potential to bridge the gap that exists between consumers and producers. Whilst consumers are more and more concerned about animal welfare and consider that it should be improved, their knowledge of farming systems is limited (Alonso *et al*. 2020; Sweeney *et al*. 2022). Consumers often do not look for details; thus, being able to back up welfare claims by providing tangible evidence and increasing trust in farm monitoring practices is important (Frewer *et al*. 2005). Similarly, being able to demonstrate that positive aspects of animal welfare are considered is particularly relevant, as animals’ affective states and ability to live ‘naturally’ are deemed important aspects by consumers when discussing animal welfare issues (Sweeney *et al*. 2022). The focus on emotions and, more generally, positive animal welfare, is also particularly relevant as it aligns with recent changes in laws relating to animal sentience, eg The Animal Welfare (Sentience) Act 2022 in the UK, which recognises that animals experience emotions that deserve consideration.

Most commercially available DLTs currently focus on minimising negative impacts on animal welfare as opposed to promoting positive experiences. This could be due to the lack of evidence regarding the validity of positive welfare indicators and the challenges in measuring them (Schillings *et al*. 2021). In addition, farmers tend to be sceptical about the use of animal-based welfare indicators (e.g. the presence of social interactions or play behaviours) and may be reluctant to adapt their systems to increase opportunities for animals to live positive experiences (Vigors & Lawrence 2019). As Vigors and Lawrence (2019) note, farmers’ approach to animal welfare is underpinned, among other things, by their values and preferences. According to a study by te Velde *et al*. (2002) farmers are not likely to actively seek to increase their knowledge about welfare and are not always aware of the importance of positive welfare aspects such as the ability to display natural behaviour. Thus, it is likely that DLTs that generate a greater understanding of different dimensions of animal welfare, as well as a reframing of values and beliefs, could lead to more meaningful impacts on management practices and animal welfare than if learning is restricted to improving strategies that are already in place (and potentially fail to address the root causes of existing issues).

In the present case studies, DLTs varied in the extent to which they fostered learning. In case study B, learning outcomes went beyond the re-visiting and improving of existing strategies that characterises single-loop learning; they led to a change in perspective and a re-questioning about what matters for animal welfare, shifting the focus from a mechanistic paradigm to one that primarily views animals as sentient beings; a process characteristic of double-loop learning. Such a perspective aligns with that of the wider public and enables farmers to communicate their process of care; that they spend more time observing their animals and considering why the animals behave in certain ways, and then where possible make improvements in management and housing that encourage positive expressions of emotional well-being (Mandel *et al*. 2016; van de Weerd & Ison 2019).

The differences in learning outcomes between the DLTs in both case studies may be related to the nature of the discussions they facilitated. In case study B, the use of the application triggered discussions and reflections around broader, emotional dimensions of animal welfare. The qualitative nature of these dimensions allows for greater flexibility in interpretation, as opposed to the more mechanistic data generated by the camera system. The interpretive flexibility of boundary objects indeed facilitates connections between different actors, and the resulting discussions can generate a better understanding of the purpose and use of a tool (Klerkx *et al*. 2012). In addition, the participative approach to the development of the application (through the generation of the terms used to conduct welfare assessments), allowed the stakeholders involved in case study B to discuss and reflect on the question of animal emotional expressivity, thus facilitating learning around this important dimension of animal welfare. By acting as boundary objects, systems developed participatively encourage social learning between stakeholders, with the level of participation having different impacts on learning (Jakku & Thorburn 2010; Reed *et al*. 2016b; Ryschawy *et al*. 2022). Participation can help bridge gaps between actors that may have different perspectives on a particular issue through discussions and feedback, allowing participants to translate different perspectives and knowledge into more concrete actions (Jakku & Thorburn 2010; van Paassen *et al*. 2011; Colnago *et al*. 2021). This is particularly relevant in the context of animal welfare, as stakeholders’ interpretation of this notion can be variable (Vanhonacker *et al*. 2008). It is therefore likely that DLTs with an interactive component may act as boundary objects and foster double-loop learning to a greater extent. In turn, this re-framing of values may lead to changes in management practices that further enhance farm animal welfare.

Previous studies have used the boundary object concept in agriculture and discussed co-learning opportunities and resulting impacts on farm management (Jakku & Thorburn, 2010; Eastwood *et al*. 2012; Morris *et al*. 2020). They have highlighted learning opportunities in promoting understanding of a concept and its values, through increased mutual understanding between actors and guidance in research and analysis (Klerkx *et al*. 2012; Duru 2013). To the authors’ knowledge, however, the concepts of single- and double-loop learning have not been used in this context in conjunction with that of boundary objects to discuss the possible extent of these objects’ impacts. The concepts of single- and double-loop learning (as well as that of triple-loop learning) were used by Reed *et al*. (2016b) to evaluate farmer learning as a result of participating in Field Labs. They highlighted the challenges in assessing changes in learning and evaluating improvements in farmers’ decision-making. Combining these concepts may thus be particularly relevant when exploring possible impacts of DLTs on learning about complex issues such as that of animal welfare, which this study aimed to do. As noted previously, animal welfare is a complex notion that can be understood differently by different people, and its improvement will depend on whether management practices will be adapted accordingly, whilst ensuring that all relevant dimensions of welfare are considered.

How DLTs impact management practices and the resulting effects on animal welfare should, however, be further explored. This includes investigating possible effects on the human-animal relationship, which is another important dimension of animal welfare (Boivin *et al*. 2003). Despite the promising potential of DLTs, previous studies have raised concerns over their potential to promote, for example, the intensification of livestock farming, or to have a negative impact on HAR if farmers were to spend less time with their animals as a result of using DLTs (Stevenson 2017; Werkheiser 2018; Schillings *et al*. 2021). In the present study, survey results suggested that whilst DLTs can assist or even replace farmers in certain welfare assessment tasks, farmers may re-direct the time saved by DLTs to other tasks which still involve human-animal interactions. This, in turn, can help improve farmers’ perceptions of the HAR and levels of welfare, particularly if those tasks assisted by DLTs were repetitive or difficult in nature. This aligns with a qualitative study by Kling-Eveillard *et al*. (2020), who found that better working conditions following the use of PLF could lead to improved human-animal relationships (HAR). Future studies should focus on the extent of these impacts from the animals’ perspectives in addition to the farmers’, combining both quantitative and qualitative methods.

## Animal welfare implications and conclusion

More sophisticated technologies are being developed with the aim of improving farmers’ working conditions as well as animal health and welfare. However, ensuring good animal welfare goes beyond the prevention of pain and illness, and includes different dimensions of welfare such as a good human-animal relationship, the ability to engage in natural behaviour and to live positive experiences. The findings of this study indicate that the impacts of using the latest artificial intelligence-based technology on animal welfare will not necessarily be greater than a simpler smartphone application. Indeed, in this study, the latter triggered deeper reflection and learning among users on important but often neglected aspects of animal welfare. Using the concepts of boundary objects and of single- and double-loop learning was a useful way to explore these impacts, by focusing on their ability to promote discussion between stakeholders, and to promote a reframing of values and beliefs. Although the benefits of smart technologies in terms of minimising negative consequences to animal health and welfare must, of course, not be ignored, this study suggests that evaluating the extent to which DLTs can help enhance farm animal welfare should also focus on their ability to encourage users to address different dimensions of animal welfare, regardless of how technologically advanced they may be.

## Supplementary material

For supplementary material accompanying this paper visit https://doi.org/10.1017/awf.2023.16. click here to view supplementary material
