# Supplementary material for: Digital Livestock Technologies as boundary objects: Investigating impacts on farm management and animal welfare
Source: Anim Welf. 2023 Feb 17;32:e17. doi: 10.1017/awf.2023.16 (PMC10936290; doi:10.1017/awf.2023.16)
Supplement: Supplementary file 1 [file awfsup.zip › S0962728623000167sup001.pdf]

Appendix 1

1. Introduction

- Research topic
- Overall aims of the study
- Confidentiality reminder
- Recording and length of interview
- Check for questions prior to start

2. General information

Aims: Background - how participants got involved in the project

- Discuss participant's role
- Start date - involvement in the project
- Aims of the project/describe technology (meaning, concept)
- Reasons for being involved

3. Experience with technology

Aims: To discuss general adoption factors and attitudes towards technology

- Describing experience with the technology

◊ Start and frequency of use

◊ Explain what it does

◊ Experience of implementation

◊ How is the data used? By whom?

◊ General impressions/attitudes (performance, ease of use, relevance…)

◊ Key factors for technology adoption

4. Participation

Aims: To uncover aspects of participation in technology development and implementation

- Involvement experience
- ◊ Extent of participation/examples

- Facilitating conditions
- ◊ Communication with other stakeholders
- ◊ Ability to provide feedback
- ◊ Feedback consideration/action
- ◊ Availability of training and support – efficiency

- Challenges and impacts
- ◊ Type of challenges met
- ◊ Have they been overcome and how
- ◊ Benefits/Drawbacks
- ◊ Attitudes
- ◊ Lessons learned

5. Impact on management and welfare

Aims: Views on the impact on learning, management and welfare

- Attitudes to technology
- ◊ Attitudes towards the concept/thoughts about the method
- ◊ Attitudes towards practicalities
- ◊ Perspectives on positive welfare and role of the technology

- Potential to promote learning
- ◊ What have they learnt about welfare
- ◊ Impact on perception understanding/knowledge of animal behaviour and welfare
- ◊ Potential for changes to welfare management
- ◊ Changes in attitudes since using the technology
- ◊ New skills acquired since the use of the technology
- ◊ Are they doing anything differently?

6. Future

Aims: Finding out their views about the future of DLTs and the wider impacts

- ◊ Use of the technology within the wider industry
- ◊ General impacts on stakeholders (farmers, consumers…)
- ◊ General impacts on animal welfare
- ◊ Any other questions

8. Conclusion

Aims: Reminding confidentiality and other aspects

- ◊ Thanks
- ◊ Confidentiality
- ◊ Contact if needed
