# Supplementary material for: Digital Livestock Technologies as boundary objects: Investigating impacts on farm management and animal welfare
Source: Anim Welf. 2023 Feb 17;32:e17. doi: 10.1017/awf.2023.16 (PMC10936290; doi:10.1017/awf.2023.16)
Supplement: Supplementary file 1 [file awfsup.zip › S0962728623000167sup002.pdf]

Appendix 2 – Survey questionnaire

Survey on Precision Livestock Farming technologies in dairy cattle farming

Please provide a **code-word** below if you would like to retain the right to withdraw your data after submission. You will need to provide us with the code-word for us to be able to identify your data and delete it.

____

Q1. What is your age category?

- ☐ 18 - 24
- ☐ 25 - 34
- ☐ 35 - 44
- ☐ 45 - 54
- ☐ 55 - 64
- ☐ 65 - 74
- ☐ 75 - 84
- ☐ 85 or above

Q2. What is your role on the farm?

- ☐ Registered business owner (land owner)
- ☐ Registered business owner (tenant)
- ☐ Farm manager (not business owner)
- ☐ Herd manager
- ☐ Other (please specify) ____________________________

Q3. In which of these regions is your farm located?

- ☐ Greater London
- ☐ South East
- ☐ South West
- ☐ West Midlands
- ☐ North West
- ☐ North East
- ☐ Yorkshire and the Humber
- ☐ East Midlands
- ☐ East of England
- ☐ Wales
- ☐ Scotland
- ☐ Northern Ireland

Q4. How many years of experience do you have in dairy farming?

☐ Less than 5 years
☐ Between 6 and 10 years
☐ Between 11 and 20 years
☐ Between 21 and 30 years
☐ Over 30 years

Q5. What is your herd size (12 months rolling herd size to include calved heifers and cows - do not include youngstock)?

Q6. What type of milking system do you use?

☐ Conventional milking parlour
☐ Automated Milking System (robot milking)

Q7. How many full-time employees are working on your dairy enterprise (including you and/or family)?

Q8. How many part-time and/or casual employees are working on your dairy?

1.1.1.1.

Q9. What type of grazing system are you operating?

☐ Housed all year round
☐ Grazed all year round
☐ Both housed and grazing periods (please indicate how many months cows are grazed per year)

In this survey, **Precision Livestock Farming** (PLF) technologies include devices such as sensors, cameras, microphones or boluses. More generally, PLF technologies are used on or around animals to help farmers monitor aspects of animal productivity, animal health and welfare or the environment, automatically and continuously.

Examples: sensors for heat detection, activity, feeding, productivity, cameras to monitor lameness or feeding, boluses to monitor rumen health, etc.

Q10. Please select which statement best describes your current situation in relation to PLF technologies:

☐ I do not currently use PLF technologies and have no intention of doing so

(if selected, please continue at Q30)

☐ I do not use PLF technologies at the moment, but I would like to use them in the future

(if selected, please continue at Q28)

☐ I am in the process of implementing one or more PLF technologies

(if selected, please continue from Q11 to Q13, then from Q23 to end of survey)

☐ I am currently using one or more PLF technologies

(if selected, please continue at Q11)

Q11. Which of the following parameters are you currently (or in the process of) monitoring using Precision Livestock Farming (PLF) technologies on your farm?

Please tick all that apply with the associated technology type.

	On-animal (wearable) sensors	Other Sensors	Camera	Bolus	Sound	Parlour/robot
Activity *(including lying and standing)*	☐	☐	☐	☐	☐	☐
Body condition	☐	☐	☐	☐	☐	☐
Body weight	☐	☐	☐	☐	☐	☐
Calving	☐	☐	☐	☐	☐	☐
Drinking behaviour	☐	☐	☐	☐	☐	☐
Environmental parameters *(temperature, ventilation...)*	☐	☐	☐	☐	☐	☐
Feeding behaviour	☐	☐	☐	☐	☐	☐
Heat	☐	☐	☐	☐	☐	☐
Lameness	☐	☐	☐	☐	☐	☐
Location	☐	☐	☐	☐	☐	☐
Mastitis	☐	☐	☐	☐	☐	☐
Methane emissions	☐	☐	☐	☐	☐	☐
Milk yield	☐	☐	☐	☐	☐	☐
Physiology *(pH, temperature...)*	☐	☐	☐	☐	☐	☐
Rumination	☐	☐	☐	☐	☐	☐
Other parameters *(please specify)*	☐	☐	☐	☐	☐	☐

Q12. If you would like to add any further details to the table above regarding the technologies you are using, please describe them below.

Q13. Please indicate how important the following factors were in your decision to adopt PLF technologies:

	Not at all important	Slightly important	Moderately important	Very important	Extremely important
Effects on productivity	☐	☐	☐	☐	☐
Health management	☐	☐	☐	☐	☐
Welfare management	☐	☐	☐	☐	☐
Ease of use	☐	☐	☐	☐	☐
Ease of installation	☐	☐	☐	☐	☐
Cost of installation	☐	☐	☐	☐	☐
Support available	☐	☐	☐	☐	☐
Address labour issues	☐	☐	☐	☐	☐
Lifestyle benefit	☐	☐	☐	☐	☐
Recommendation by advisors/peers	☐	☐	☐	☐	☐
Environmental benefits	☐	☐	☐	☐	☐
Other (*please specify*)	☐	☐	☐	☐	☐

Q14. What kind of changes to your farm management routines resulted from the use of PLF technologies, if any?

	No changes	Minor changes	Major changes
Routine tasks	☐	☐	☐
Work schedule	☐	☐	☐
Number of full-time staff	☐	☐	☐
Number of part-time/casual staff	☐	☐	☐
Time spent on digital devices (computers, tablets, phones)	☐	☐	☐
Time spent with animals	☐	☐	☐
Other (*please specify*)	☐	☐	☐

Q15. How much do you or your staff rely on PLF technologies to monitor your herd in comparison to visual or manual monitoring, across all management tasks?

Please cross the bar under the appropriate percentage. For example, if you rely on technology and manual monitoring equally, cross the bar under '50'.

Not relying on PLF at all					50/50					Complete reliance on PLF
0	10	20	30	40	50	60	70	80	90	100%

____

Q16. How often do you manually or visually verify whether the data collected by the technologies are accurate? *In the case of heat detection for example, by looking for additional, visual signs of cows in heat.*

☐ Never

☐ Sometimes

☐ About half the time

☐ Most of the time

☐ Always

Q17. How has the time spent visually or manually assessing the health and welfare of your herd changed since the implementation of PLF technologies?

☐ Substantially decreased

☐ Somewhat decreased

☐ About the same

☐ Somewhat increased

☐ Significantly increased

Q18. How has human contact with the cows in the herd changed since the implementation of PLF technologies?

☐ Substantially decreased

☐ Somewhat decreased

☐ About the same

☐ Somewhat increased

☐ Significantly increased

Q19. In your opinion, how is the relationship between stockpeople and the herd since the implementation of the technology?

☐ Much worse

☐ Somewhat worse

☐ About the same

☐ Somewhat better

☐ Much better

Q20. How did the technologies affect the parameters they are designed to monitor?

For example, improved reproduction with heat detection or reduction of lameness prevalence in the case of automatic lameness monitoring.

	Much worse	Somewhat worse	About the same	Somewhat better	Much better
Technology 1: ______________ (*Please indicate which technology you are referring to*)	☐	☐	☐	☐	☐
Technology 2: _______________	☐	☐	☐	☐	☐
Technology 3: _______________	☐	☐	☐	☐	☐
Technology 4: _______________	☐	☐	☐	☐	☐
Technology 5: _______________	☐	☐	☐	☐	☐

Q21. In your opinion, how has the welfare of animals in your herd changed due to the implementation of PLF technologies?

☐ Much worse

☐ Somewhat worse

☐ About the same

☐ Somewhat better

☐ Much better

Q22. In your opinion, how has the behaviour of your livestock changed since the implementation of PLF technologies?

	They are more...	They are less...	No change
Relaxed	☐	☐	☐
Calm	☐	☐	☐
Content	☐	☐	☐
Friendly	☐	☐	☐
Nervous	☐	☐	☐
Indifferent	☐	☐	☐
Distressed	☐	☐	☐
Uneasy	☐	☐	☐

Q23. In your opinion, how effective was the training you have received to efficiently use the technology?

☐ Not effective at all

☐ Slightly effective

☐ Moderately effective

☐ Very effective

☐ Extremely effective

☐ Not applicable

Q24. What kind of challenges, if any, have you encountered during the implementation of PLF technologies on your farm?

Q25. If you have encountered any challenges, please describe whether these have been overcome and if so, how?

Q26. If you have encountered any challenges, how have they affected your attitudes towards the technologies?

☐ Very negative impact

☐ Slightly negative impact

☐ No impact

☐ Slightly positive impact

☐ Very positive impact

☐ Not applicable

Q27. To what extent do you agree with the following statements in relation to PLF technologies on your farm?

	Strongly disagree	Somewhat disagree	Neither agree nor disagree	Somewhat agree	Strongly agree	Not applicable
I was able to provide feedback to technology providers	☐	☐	☐	☐	☐	☐
Technology providers have helped me interpret the data efficiently	☐	☐	☐	☐	☐	☐
Technology providers are transparent regarding ownership and use of data	☐	☐	☐	☐	☐	☐
I believe my feedback is taken into account in the development of the technologies	☐	☐	☐	☐	☐	☐
Challenges met during implementation have been actively addressed by the technology providers	☐	☐	☐	☐	☐	☐

Q28. What has prevented you from implementing PLF technologies on your farm so far? *Please provide examples where possible.*

Q29. Which types of PLF technologies would you like to implement in the future and why?

Q30. What are the reasons why you do not wish to implement PLF technologies on your farm?
